# Postoperative radiotherapy in the management of keloids

**DOI:** 10.3332/ecancer.2016.690

**Published:** 2016-11-08

**Authors:** Claudia C Carvajal, Carla M Ibarra, Douglas L Arbulo, Moisés N Russo, Claudio P Solé

**Affiliations:** 1 Servicio de Radioterapia, Clínica IRAM, Santiago 7630595, Chile.; 2 Servicio de Cirugía General, Hospital Militar, Santiago, Chile.; 3 Service de Radiothérapie, Institut Gustave Roussy, París 94805, France.

**Keywords:** keloid, surgery, radiotherapy

## Abstract

**Background:**

The high recurrence rate following keloid resection has generated interest in adjuvant treatments for this disease.

**Objective:**

This study assesses keloid recurrence when treated with surgery and adjuvant radiotherapy.

**Methods:**

Retrospective analysis of resected keloids in patients referred to a Chilean radiation oncology centre between 2006 and 2013. Local recurrence was defined as new tissue growth on the surgical scar margin.

**Results:**

Around103 keloids were analysed in 63 patients treated with 15 Gy in three fraction radiotherapy which was initiated on the same day as the surgery (75% of cases). The median keloid diameter was 6 cm; the most common site was thoracic (22%); the most common cause was prior surgery (35%); 37% caused symptoms, and several (47%) had received prior treatment with corticosteroids (32%), or surgery (30%). The median follow-up was three years, and 94% of recurrences occurred during the first year following treatment. Uni and multivariate analyses showed that an absence of symptoms was a protective factor for recurrence (OR: 0.24), while the time interval from onset to treatment with surgery plus radiotherapy >4.2 years was a risk factor (OR: 2.23). The first year recurrence rate was 32% and stabilised at 32% by the second year with no recurrences after 15 months.

**Conclusions:**

The combination of surgery and radiotherapy proved to be a good therapeutic alternative in the management of keloids. Our results are similar to those described in the literature for a dose of 15 Gy. Given these results, our centre will implement a new dose escalation protocol to improve future outcomes.

## Background

A keloid is a neoplasic pathological scar which extends beyond the margin of the original incision. They originate from various skin lesions such as surgery, burns, acne, or may appear spontaneously. They are more common in females and tend to be located on the upper body; primarily on the face, ears, and thorax. They can cause various symptoms such as pain, itching, or inflammation, such that in many cases they represent not only a cosmetic change but also a functional one.

Simple surgical resection of this type of lesion has a local recurrence rate greater than 50% [1], and for this reason multiple attempts have been made to find adjuvant therapies which can increase the success rate of treatment. Some available postoperative therapies are: radiotherapy, silicone bandaging, compression bandaging, cyrosurgery, and intralesional injections (corticosteroids, 5-fluorouracil, verapamil, bleomycin) among others.

At present there is no consensus on the best treatment for the management of keloids, and this is mainly because of the fact that the published evidence consists primarily of retrospective studies [2]. Nevertheless, it is important to point out that these studies consistently favour combined therapies over unimodal ones [1, 3]. Radiotherapy has stood out from the other alternative adjuvant therapies because of its good tolerance [4–5], also the fact that it is non-invasive and requires less time than other therapies for administration and achievement of a significant reduction in recurrences when it is added postoperatively.

Treatment with surgery plus radiotherapy in the multimodal management of keloids requires a multidisciplinary team which involves both the surgical team and the radiation oncology team during the evaluation, treatment, and follow-up stages.

This is the first Chilean study published on the management of this disease. This study was conducted under the hypothesis that a retrospective review of the results at a single institution could provide a basis for adjusting the total dose of adjuvant radiotherapy to be given to future patients, as several international studies have proposed new dosing fractions in order to further reduce recurrences.

The main objective of this study was to calculate the recurrence rate of this benign tumour following multimodal therapy with surgery plus external radiotherapy in a Chilean institution which receives patients who have had surgery in multiple centres in the country which have a common therapeutic focus.

## Methods

A retrospective analysis was done of the clinical files of all patients receiving electron radiation treatment in a radiation oncology centre. The patients were referred from different surgical centres to receive adjuvant radiotherapy during the years 2006–2013.

The surgical technique for excising a keloid consists of resecting the lesion at its margins without leaving the lesion *in situ*, and suturing at several levels from the base to the surface with the goal of placing more tension on the subcutaneous cellular tissue in order to promote closure of the skin. A surgery is considered successful if it achieves a scar with well-defined margins which is linear and without tension.

Postoperative radiotherapy uses a computed axial tomography (CAT) scan on which the surgical wound bed and the organs at risk are outlined. With the help of a treatment planning system which simulates the penetration of the electrons into the area outlined, the linear accelerator is programmed to deliver the prescribed dose in order to control the excessive proliferation of fibroblasts in the surgical wound bed.

The following data were retrieved from the files: file number, patient name, year of admission, age, sex, national identification number, telephone, referring centre, location of the keloid, cause of the lesion, symptomatology, date of first appearance of the keloid, prior use of corticosteroids, number of prior surgeries, maximum diameter of the lesion, date of resection, dates of the first and last radiotherapy sessions, total time and dose of radiotherapy, energy of the electrons, use of a bolus and its thickness, length and width of the radiotherapy field, inclusion of the thyroid and mammary beds in the radiotherapy field, referring surgeon, and date of last clinical visit and/or telephone consultation.

The statistical analysis was performed using the SPSS programme version 19.0 to calculate the rate of recurrence using Kaplan-Meier tracking measurement curves, and possible protective or risk factors were identified through the logistic regression (Odds Ratio) model.

The protocol for this investigation was reviewed and approved by the ethics committee of the Faculty of Medicine of Diego Portales University.

## Results

**Figure 1.**
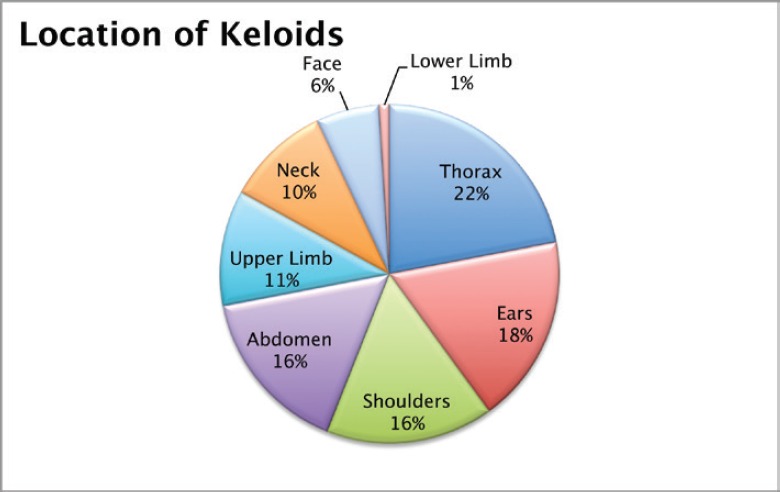
Location of the keloids.

**Figure 2.**
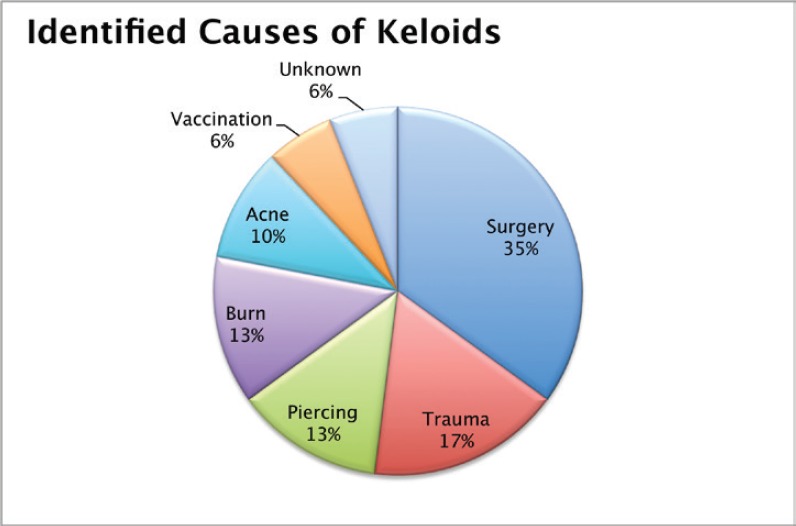
Causes identified for the keloids.

A total of 103 keloids were analysed in 63 patients with a median age of 30 years [range 13–77]. This series represents the full experience of a Chilean radiation oncology centre with keloids resected and irradiated with electrons as all of the patients who were treated in this manner between the years 2006 and 2013 were included. One patient was excluded from the analysis because of his premature death from another cause (subarachnoid haemhorrage). Among these 54% of the patients were men. Most of the patients (61%) had multiple keloids which were treated simultaneously. More details of patient characteristics can be seen in Table 1. The keloids included those resected by 24 plastic surgeons from different centres in Chile. The median time between the appearance of the keloid and the start of surgical treatment plus radiotherapy was 4.1 years [range 2–299 months]. Radiotherapy was given in a dose of 15 Gy in three fractions with electrons from 4.5 to 10 meV, except for one case which received 16 Gy in four fractions without any special risk factors. A bolus of 0.5–1 cm was used for 98% of the lesions. Adjuvant treatment with radiotherapy was started the same day as surgery in 75% of the cases [range of 0–31 days]. The median diameter of the lesions was 6 cm [range 1–34] and that of the field size was 7 cm [range 4–28]. The locations of the lesions were grouped as follows: face, ears, neck, shoulders, upper limbs, thorax, abdomen, and lower limbs. The percentages in which these were distributed can be seen in detail in Figure 1. The original causes of the keloids can be seen in Figure 2 grouped by: surgery, trauma, piercings, burns, acne, vaccinations, or unknown. The reason for seeking treatment was pain or itching in 37% of the keloids, and in the rest of the lesions did not cause symptoms. Around 32% of the keloids had received prior treatment with corticosteroids and 30% with surgery. There was no difference in the response of the keloids with or without treatment prior to surgery plus radiotherapy. Seven treatment fields had contact with the thyroid or breast bed on some of their margins. A total 35% of the lesions had recurred by the time of the telephone interview, and six lesions were not followed up on. The median follow-up was 3.35 years [range 12.3–85.6 months] and the great majority of recurrence (94%) became evident during the first year following treatment.

**Figure 3.**
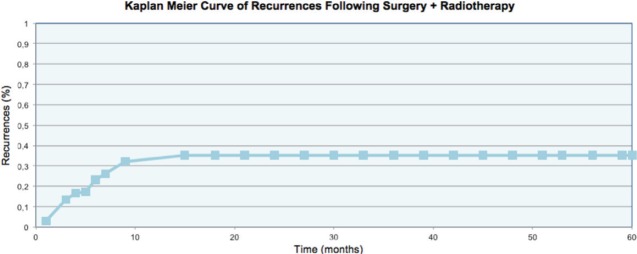
Recurrence rate of the keloids.

The data was analysed using the linear regression model. The uni and multivariate analyses demonstrated that the absence of symptoms prior to treatment constituted a protective factor for recurrence (OR:0.24), while a time between appearance of the keloid and treatment with surgery plus radiotherapy greater than 4.5 years was a risk factor (OR:2.23) for recurrence. More details of the uni and multivariate analyses are included in Tables 2 and 3. The rate of recurrence at one year was 32%, and at two, three, four, and five years stabilised to 35%, as can be seen in Figure 3. One patient was diagnosed with bladder cancer during the follow-up, but his field of radiotherapy did not include this organ.

## Discussion

This retrospective series of keloids is the first to be published on Chilean patients receiving surgery and radiation. Prior to this publication, the national literature included the review of Andrades *et al* [6], who proposed among their flow charts the use of adjuvant radiotherapy to improve postsurgical local control in the management of keloids.

Many of the patients came to their treatment with surgery plus radiotherapy after having received other prior unimodal treatments such as corticosteroids and surgery without success. The wide variety of treatments that are applied to keloids can be attributed, among other things, to the fact that most of the published evidence on the management of this disease is composed of institutional reports whose results are difficult to compare and interpret. Our proposal, faced with the lack of a randomised prospective study which compares surgery only with surgery plus radiotherapy, is to base our new recommendations on the prospective studies which will explicitly define the event ‘recurrence’ as the primary objective.

Previous retrospective studies which reported good results with surgery plus adjuvant radiotherapy, promoted a total dose which ranged between 10 and 20 Gy. For this reason the oncology centre in which the patients from this Chilean series received treatment were administered 15 Gy in three fractions of adjuvant radiotherapy during the three days following surgery. Some examples of these studies are those of Pérez *et al* [7–8], who in their final report showed a recurrence rate of 33% using a total dose between 10 and 20 Gy, identical to that obtained by the group of Darzi *et al* [3] with 16 Gy in four fractions. Ragoowanski *et al* [9] used a single full fraction of 10 Gy and achieved a recurrence rate of 16%. In Germany, Kutzner *et al* [10] also used total doses between 10 and 20 Gy and achieved a failure rate of 11.4%. Doornbos *et al* [11] suggested in their review that a dose lower than 9 Gy not be used because of its low effectiveness and posited that with total doses greater than 15%, the recurrences would be reduced to less than 10%. Shen *et al* [12] published their results with 834 keloids treated with electrons in two full fractions to achieve a total dose of 18 Gy with a failure rate of 9.6%.

If we analyse the results of the prospective series, these seem to support the findings of the previous studies that the higher the total dose of adjuvant radiotherapy, the lower the rates of recurrence and vice versa. For example, the work of Ogawa [13] showed a recurrence rate of 29.3% using 15 Gy, which could be reduced to 14% when a larger total dose of 20 Gy was administered to certain lesions. Another study which supports the results obtained by Ogawa is that of Kuribayashi *et al* [14], who achieved a recurrence rate of 9.7% with brachytherapy using a total dose of up to 20 Gy. The opposite was shown in the series by van de Kar *et al* [15], who used a small total dose of 12 Gy and achieved a high recurrence rate (71.9%).

Although radiotherapy is not the only adjuvant treatment available for the management of keloids, it has substantial advantages over other alternatives because of the fact that these tend to require long periods of time for completion. Sclafani *et al* [4] compared radiotherapy with postoperative intralesional corticosteroids and observed greater effectiveness and comfort for those patients receiving additional radiotherapy. The same occurred years later in the study by Emad *et al* [5], which compared cryotherapy associated with corticosteroids to radiotherapy.

According to the literature review by Flickinger [16], the rates of recurrence can be lower than 10% if total doses greater than 16 Gy are given with adjuvant radiotherapy.

The radiobiological differences between the same total dose divided into different fractioning plans (one versus several fractions) can be discussed at length, but this question is not among the objectives of this discussion. Nevertheless, it is worth pointing out the review by Kal *et al* [17], who analysed multiple fractioning plans and concluded that biologically effective doses (BEDs) greater than 30 Gy should achieve failure rates below 10% (Table 4).

With respect to the fear which exists that radiotherapy can induce secondary effects such as delayed toxicity, we observe from our report that in our series only one patient developed a malignant neoplastic disease during the follow-up time after keloid treatment, but this did not occur within the radiotherapy field. Of course, the follow-up time and the number of patients make it impossible to draw greater conclusions. With respect to acute toxicity, the most likely to occur in these patients is either cutaneous erythema or radiodermatitis. The details of this though cannot be reported as not all patients had toxicity reports included in their follow-up.

It is regrettable that we cannot report on the cosmetic results because of the lack of application of an objective scale. An important characteristic to include in future studies is the use of a validated scar reporting scale which would permit the standardisation of the results measured during the clinical follow-up, such as the Patient And Observer Scar Assessement Scale (POSAS) [18] for example.

Another important observation which can be made from our results as well as those in the literature is that when the treatment fails, the majority of recurrences occur within the first year and only 10% appear during the second year of follow-up. Thus the definitive result can be estimated with good certainty in the medium term. The rate of recurrence at two years was 35%, hence placing our study at the high end of the expected range of recurrences for a total dose of 15 Gy.

Finally, given that current reviews propose an increase in the total dose to 21–30 Gy with recurrence rates significantly lower than those obtained with lower doses, the results shown in this article plus a review of the literature caused a change in protocol in the oncology centre in which this study was done. A new protocol was initiated with a dose of 21 Gy in three fractions with the goal of reducing the recurrence rate of keloids managed with surgery plus adjuvant radiotherapy. The results of said series will be stored in a prospective manner with the additional application of a validated POSAS [18] scale by the observer, and the patient along with these will be reported on in the future.

## Conclusions

In our experience, the addition of radiotherapy following surgical resection of keloids was a good therapeutic alternative with similar results to those described in the literature for the total dose which was used. This performance could be increased by using higher doses of adjuvant radiotherapy.

## Conflicts of interest

The authors deny the existence of any conflicts of interest.

## Source of financial support

None.

## Figures and Tables

**Table 1.** Characteristics of the patients and their keloids (n = number).

PATIENT CHARACTERISTICS		n	%
**Sex**	Male	55	53
	Female	48	47
**no. of keloids per patient**	Single	23	39
	Multiple	80	61
**Cause**	Piercing	13	13
	Surgery	36	35
	Vaccination	6	6
	Burn	14	13
	Acne	10	10
	Trauma	18	17
	Unknown	6	6
**Symptoms**	Yes	38	37
	No	65	63
**Prior treatments**	Surgery	31	30
	Corticosteroids	33	32
**Field over breast or thyroid**	Yes	7	7
	No	96	93

**Table 2.** Univariate analysis with linear regression model (n = number, CI = Confidence Interval, OR = Odds Ratio).

UNIVARIATE ANALYSIS	Variable	Recurrence-free survival		
		OR	CI 95%	p value
**Age**	≤30 years	1,0	0,36–2,08	0,79
	>30 years	0,89		
**Sex**	Male	1,0	0,63–3,37	0,38
	Female	1,46		
**No. of keloids**	Single	1,0	0,94–5,31	0,07
	Multiple	2,23		
**Appearance**	Primary	1,0	0,55–3,71	0,48
	Recurrence	1,43		
**Symptoms**	Yes	1,0	0,09–0,69	0,008
	No	0,25		
**Keloid diameter**	>5 cm	1,0	0,24–1,35	0,2
	≤5 cm	0,58		
**Location**	Limbs	1,0		
	Head and Neck	1,05	0,53–2,56	0,68
	Trunk	1,02	0,62 –1,16	0,95
**Interval from diagnosis to**	≤50 months	1,0	1,0 –4,93	0,04
**Surgery+radiotherapy**	>50 months	2,07		

**Table 3.** Multivariate analysis with linear regression model (CI = Confidence Interval, OR = Odds Ratio).

MULTIVARIATE ANALYSIS	Variable	Recurrence-free survival		
		OR	CI 95%	p value
**Symptoms**	Yes	1	0,09–0,68	0,007
	No	0,24		
**Interval from diagnosis to**	≤50 months	1	1,01–5,44	0,05
**Surgery+radiotherapy**	>50 months	2,23		

**Table 4.** Retrospective/prospective studies and their comparison according to BED.

	Total Dose (Gy)	Number of fractions	BED α/β = 3 (Gy)	BED α/β = 10 (Gy)	Recurrence (%)	Years of follow-up (median)
**RETROSPECTIVE STUDIES**						
Pérez *et al* (7)	12	3	19	11	33	6.75 (median)
Darzi *et al* (3)	16	4	37	22	33	
Ragoowansi *et al* (9)	10	1	43	20	16	
Kutzner *et al* (10)	10–20	5–10	17–33	12–24	11,4	
Doornbos *et al* (11)	6–15	3	10–40	7–23	23,5	
Shen *et al* (12)	18	2	72	34	9,6	3.3 (median)
**PROSPECTIVE STUDIES**						
Ogawa *et al* (13)	10–20	2–4	27–53	15–30	14	1.9 (median)
Kuribayashi *et al* (14)	15–20	3–4	40–53	23–30	9,7	1.5 (median)
van de Kar *et al* (15)	12	3–4	28–24	17–16	71,9	1.5 (median)
Sclafani *et al* (4)	7–10	1	23–43	12–20	12,5	1.5 (median)
Emad *et al* (5)	12	3	28	17	18,2	1.5 (median)

## References

[1] Cosman B (1961). The surgical treatment of keloids. Plast Reconst Surg.

[2] Leventhal D, Furr M, Reiter D (2006). Treatment of keloids and hypertrophic scars: a meta-analysis and review of the literature. Arch Facial Plast Surg.

[3] Darzi MA (1992). Evaluation of various methods of treating keloids and hypertrophic scars: a 10-year follow-up study. Br J Plast Surg.

[4] Sclafani AP (1996). Prevention of earlobe keloid recurrence with postoperative corticosteroid injections versus radiation therapy: a randomized, prospective study and review of the literature. Dermatol Surg.

[5] Emad M (2010). Surgical excision and immediate postoperative radiotherapy versus cryotherapy and intralesional steroids in the management of keloids: a prospective clinical trial. Med Princ Pract.

[6] Andrades P, Benitez S, Prado A (2006). Recomendaciones para el manejo de cicatrices hipertróficas y queloides. Rev Chil Cir.

[7] Perez CA, Lockett MA, Young G (2001). Radiation therapy for keloids and plantar warts. Front Radiat Ther Oncol.

[8] Kovalic J, Perez CA (1989). Radiation therapy following keloidectomy: a 20-year experience. Int J Radiat Oncol.

[9] Ragoowansi R (2003). Treatment of keloids by surgical excision and immediate postoperative single-fraction radiotherapy. Plast Reconstr Surg.

[10] Kutzner J (2003). Radiotherapy of keloids. Patterns of care study–results. Strahlenther Onkol.

[11] Doornbos J (1990). The role of kilovoltage irradiation in the treatment of keloids. Int J Radiat Oncol.

[12] Shen J (2015). Hypofractionated electron-beam radiationtherapy for keloids: retrospective study of 568 cases with 834 lesions. J Radiat Res.

[13] Ogawa R (2007). Postoperative radiation protocol for keloids and hypertrophic scars: statistical analysis of 370 Sites followed for over 18 Months. Ann Plast Surg.

[14] Kuribayashi S (2011). Post-keloidectomy irradiation using high-dose-rate superficial brachytherapy. J Radiat Res (Tokyo).

[15] Van de Kar AL (2007). The results of surgical excision and adjuvant irradiation for therapy-resistant keloids: a prospective clinical outcome study. Plast Reconstr Surg.

[16] Flickinger JC (2011). A radiobiological analysis of multicenter data for postoperative keloid radiotherapy. Int J Radiat Oncol.

[17] Kal HB, Veen RE, Jürgenliemk-Schulz IM (2009). Dose–effect relationships for recurrence of keloid and pterygium after surgery and radiotherapy. Int J Radiat Oncol.

[18] Nicholas RS (2012). Patient-related keloid scar assessment and outcome measures. Plast Reconstr Surg.

